# Infant traumatic brain injury with a biphasic clinical course and late diffusion restriction: a case report

**DOI:** 10.3389/fnins.2026.1760034

**Published:** 2026-05-20

**Authors:** Dong He, Rong Yan, Peng Zhang, Xiang Yue, Shunwu Xiao

**Affiliations:** 1 Department of Neurosurgery, Affiliated Hospital of Zunyi Medical University, Zunyi, Guizhou, China; 2 Department of Neurology, Affiliated Hospital of Zunyi Medical University, Zunyi, China

**Keywords:** biphasic clinical course, excitotoxicity, infant traumatic brain injury, late diffusion restriction, magnetic resonance imaging

## Abstract

Traumatic brain injury (TBI) in young children can rarely exhibit a biphasic clinical course with delayed neurological deterioration. We report a 2-year-old boy who fell from 50 cm and briefly lost consciousness with vomiting, initially found to have a right frontotemporoparietal acute subdural hematoma (SDH) with midline shift but no brain contusions. After transient stabilization, he developed new left-sided limb weakness and status epilepticus on day 3 post-injury. Follow-up diffusion-weighted magnetic resonance imaging (DWI) revealed a characteristic “bright tree” pattern of bilateral subcortical white matter diffusion restriction with corresponding decreased apparent diffusion coefficient (ADC) values. Electroencephalography showed generalized slowing with interictal focal epileptiform discharges. The patient was managed with antiepileptic therapy and supportive care. He demonstrated steady improvement and achieved near-complete neurological recovery by 9-month follow-up. This biphasic presentation—early trauma and late-onset seizures with diffusion restriction—is consistent with Traumatic brain injury with a biphasic clinical course and late reduced diffusion (TBIRD). Early recognition of TBIRD is crucial, as it resembles acute encephalopathy with biphasic seizures and late diffusion changes, and likely stems from secondary excitotoxic injury. Timely intervention in our case was associated with a favorable outcome, underscoring the importance of vigilant monitoring for delayed neurologic sequelae in pediatric TBI.

## 1 Introduction

Traumatic brain injury with a biphasic clinical course and late reduced diffusion (TBIRD) is a distinct subtype of pediatric TBI. Clinically, it is characterized by initial neurological impairment immediately following trauma, followed by secondary deterioration several days later. During the subacute phase, diffusion-weighted imaging (DWI) reveals a characteristic “bright tree appearance” indicative of restricted diffusion in the subcortical white matter (Takase et al., 2018). These clinical and radiological features resemble those of acute encephalopathy with biphasic seizures and late reduced diffusion (AESD), a common infection-induced encephalopathy in children characterized by febrile seizures (Nagai et al., 2025). However, TBIRD is distinguished by its traumatic etiology; its core symptomatology involves status epilepticus and is frequently associated with subdural hematoma (SDH) (Kawano et al., 2025). The exact pathogenesis of TBIRD remains elusive. However, prevailing theories focus on excitotoxicity and metabolic dysregulation. Following head trauma, an excessive release of glutamate overactivates N-methyl-D-aspartate (NMDA) and *α*-amino-3-hydroxy-5-methyl-4-isoxazolepropionic acid (AMPA) receptors, triggering a cascade of neuronal injury and delayed cell death (Hoffe and Holahan, 2022; Bell et al., 2009). At present, its clinical management still mainly relies on empirical supportive treatment, including antioxidant therapy, therapeutic hypothermia, and seizure control (Yasukohchi et al., 2024a,b). As a rare syndrome occurring after head trauma in infants and young children, TBIRD may lead to persistent neurological deterioration in some patients. Therefore, early recognition is of great importance for disease management. Previous reports have shown that most documented cases of TBIRD occurred in infants younger than 1.5 years of age (Yasukohchi et al., 2024a,b; Hori et al., 2023). Here, we present a detailed report of a 2-year-old child who developed TBIRD after a head injury and subsequently achieved a good recovery following aggressive anticonvulsant treatment and adjunctive neurotrophic supportive therapy. This study aims to improve clinical awareness of TBIRD and to provide a reference for its early recognition and management.

## 2 Case report

A 2-year-old boy was admitted to our hospital 2 h after a head injury. The patient had fallen approximately 50 cm from a sofa, striking the occipital region. He immediately cried, vomited, and experienced brief bilateral upper limb convulsions that lasted a few seconds before resolving. On admission, the patient’s vital signs, including body temperature, pulse rate, respiratory rate, and blood pressure were within normal limits. Neurological examination revealed somnolence, with a Glasgow Coma Scale score of E4V3M5. Both pupils were equal and round, measuring approximately 2.5 mm in diameter, with brisk light reflexes. Spontaneous limb movements were observed in response to painful stimuli, muscle tone was normal, and pathological reflexes were negative. Other examinations were not feasible due to poor cooperation. Computed tomography (CT) of the head revealed an acute subdural hematoma (ASDH) over the right frontotemporoparietal region with midline shift but no evidence of cerebral contusion (Figure 1A). The patient’s parents declined surgical intervention; therefore, conservative medical management was initiated. To prevent seizure occurrence, intravenous sodium valproate was administered (0.3 g sodium valproate in 47 mL of 0.9% sodium chloride solution at 2 mL/h). A follow-up head CT performed on hospital day 2 showed a reduction in the hematoma size (Figure 1B). At that time, the patient’s vital signs were stable, consciousness was clear, muscle strength and tone in all extremities were normal, tendon and physiological reflexes were preserved, and no pathological reflexes were elicited. As no further seizures occurred and both the clinical condition and repeat head CT findings had improved, intravenous sodium valproate was switched to oral sodium valproate (3 mL, twice daily) for seizure prophylaxis.

**Figure 1:**
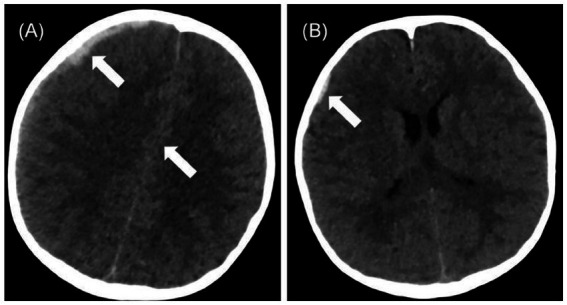
Cranial computed tomography (CT) scans of the patient. **(A)** CT on admission revealed a right frontoparietal subdural hematoma with a leftward midline shift (arrow). **(B)** Follow-up CT within 24 h after admission showed a reduction in the size of the right frontoparietal subdural hematoma (arrow).

On the third day, the patient developed decreased muscle strength on the left side, manifested as grade 0 strength in the left upper limb, grade 4 strength in the left lower limb, and grade 5 strength in the right extremities, while muscle tone remained normal. The tendon reflex of the left upper limb could not be elicited, whereas tendon reflexes in the other extremities were normal. No pathological reflexes were observed. In view of these newly developed neurological deficits, repeat cranial imaging was performed. Cranial CT revealed a low-density lesion in the occipital lobe. Brain magnetic resonance imaging (MRI) showed hyperintensity on DWI in the right cerebral hemisphere and the left frontoparietal-occipital white matter, presenting a “bright tree” appearance (Figure 2A). The apparent diffusion coefficient (ADC) maps demonstrated reduced values in the affected white matter (Figure 2B), whereas fluid-attenuated inversion recovery (FLAIR) images showed no obvious abnormality in white matter signal intensity (Figure 2C). Based on the patient’s clinical course and characteristic MRI findings, the diagnosis of TBIRD was confirmed. Considering the similarities in pathophysiological mechanisms between TBIRD and AESD, as well as our previous clinical experience, vitamin B6 was added to the ongoing sodium valproate therapy for seizure prophylaxis as an adjunctive treatment to mitigate excitotoxicity.

**Figure 2:**
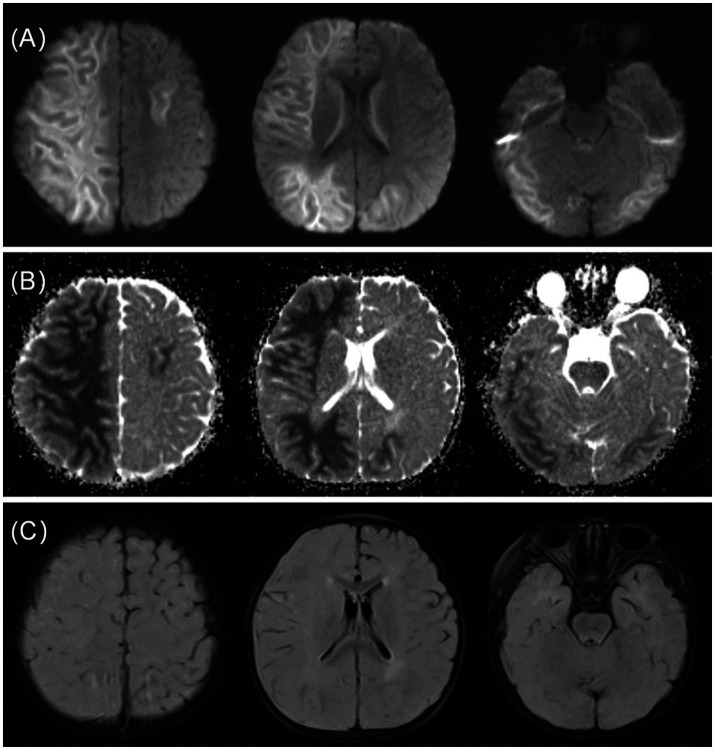
MRI performed on day 3 demonstrated a bright, tree-like appearance in the right frontal, parietal, and occipital lobes on DWI. The lesion showed low signal intensity on ADC map and isointensity on fluid-attenuated inversion recovery (FLAIR) imaging. **(A)** DWI; **(B)** ADC map; **(C)** FLAIR image.

On the fourth day, the patient developed cluster seizures involving the left limbs, accompanied by impaired consciousness, upward eye deviation, trismus, and generalized tonic stiffness. Each episode lasted approximately 2–3 min and occurred four times in total, during which the patient remained unconscious between seizures. Electroencephalographic (EEG) monitoring revealed generalized slow-wave activity dominated by *δ* waves (Figure 3). Even during the interictal period, focal epileptiform discharges originating from the right anterior temporal region were detected. Based on the clinical and EEG findings, the patient was diagnosed with status epilepticus. Oral sodium valproate was discontinued, and continuous intravenous infusion of sodium valproate (0.3 g sodium valproate diluted in 27 mL of 0.9% sodium chloride solution, at 1.5 mL/h) was initiated in combination with diazepam (10 mg diazepam diluted in 50 mL of 5% glucose solution, at 2–6 mL/h). On the fifth day, the patient experienced no further seizures, and diazepam injection was discontinued. Neurological examination revealed muscle strength of grade 1 in the left upper limb and grade 3 in the left lower limb, with the remainder of the examination unremarkable. Thereafter, treatment was mainly focused on maintaining antiseizure therapy and reducing excitotoxicity. On the thirteenth day, follow-up MRI revealed a marked reduction in the abnormal signal intensity within the subcortical white matter of the right cerebral hemisphere and the left fronto-parieto-occipital lobes on DWI (Figure 4A). However, the fluid-attenuated inversion recovery (FLAIR) images showed a hyperintense area accompanied by atrophy in the right cerebral hemisphere (Figure 4B). On the sixteenth day, neurological examination revealed muscle strength of grade 4 in the left upper limb, with no other abnormalities detected. The patient’s Pediatric Cerebral Performance Category (PCPC) score was 2. After comprehensive clinical evaluation, the patient was discharged. At the 9-month follow-up, the patient’s development was normal, the PCPC score was 1, and no neurological deficits were observed.

**Figure 3:**
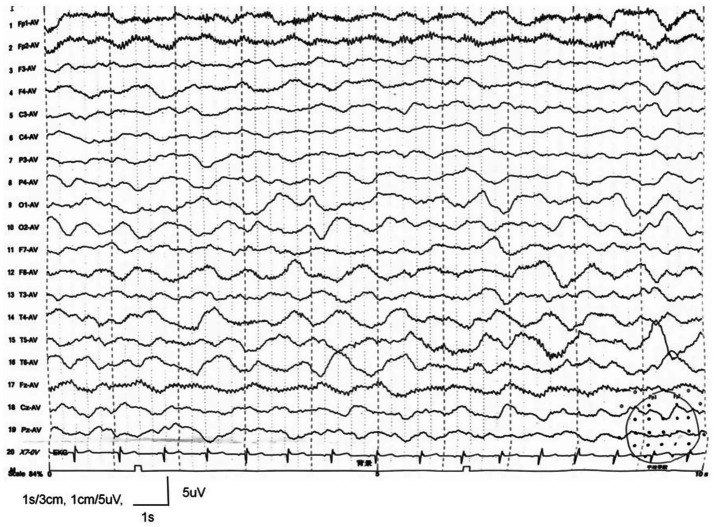
Continuous 24-h video electroencephalogram (EEG) monitoring results. The EEG showed generalized slowing, predominantly characterized by delta wave activity.

**Figure 4:**
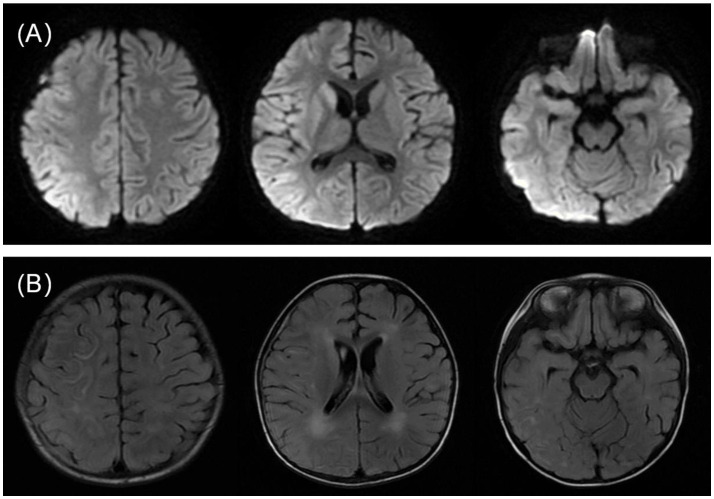
MRI obtained on day 13 shows a marked reduction in abnormal signal intensity in the right cerebral hemisphere and the subcortical white matter of the left frontoparietal–occipital lobes on DWI. However, fluid-attenuated inversion recovery (FLAIR) imaging reveals an atrophic hyperintense area in the right cerebral hemisphere. **(A)** DWI. **(B)** FLAIR image.

## 3 Discussion

TBI is defined as injury to the brain parenchyma caused by external mechanical force. Whether occurring in isolation or concomitantly with multisystem trauma, TBI remains a severe neurological disorder characterized by high mortality and disability rates (Popescu et al., 2015). Recently, a biphasic clinical course has been observed in certain infants and young children following TBI. In these patients, cranial DWI reveals delayed restricted diffusion manifesting as a characteristic “bright tree-like” appearance, which is frequently associated with long-term neurological deficits. This distinct entity is termed TBIRD, a condition sharing pathological and radiological similarities with AESD (Takase et al., 2018). The pathogenesis of TBIRD remains not fully elucidated. Metabolic imaging studies using magnetic resonance spectroscopy (MRS) indicate that patients with TBIRD exhibit distinct metabolic abnormalities compared to non-TBIRD counterparts, characterized primarily by significantly elevated glutamine (Gln) levels and reduced N-acetylaspartate (NAA) concentrations. Specifically, Gln levels peak between days 3 and 29 post-injury before gradually normalizing, whereas the extent of NAA reduction correlates positively with disease severity (Yasukohchi et al., 2024a,b). A similar metabolic pattern has been reported in AESD, which features glutamate (Glu) elevation during the acute phase followed by a shift toward elevated Gln in the subacute phase (Takanashi et al., 2015).

Seizures constitute a core symptom of TBIRD. Research suggests that prolonged early seizures (>30 min), the requirement for tracheal intubation, and acute CT findings of midline shift or unilateral cerebral edema are significant risk factors for the progression of TBI to TBIRD (Yasukohchi et al., 2024a,b). Pathophysiologically, epilepsy involves abnormal, hypersynchronous neuronal discharge resulting from an imbalance between excitatory and inhibitory neurotransmission (Paz and Huguenard, 2015). Under normal physiological conditions, glutamate—the primary excitatory neurotransmitter—is sequestered by astrocytes via Na^+^-dependent transporters and converted into Gln by glutamine synthetase. Gln is subsequently shuttled back to neurons and reconverted to Glu, thereby maintaining neurotransmitter homeostasis (Sheldon and Robinson, 2007). However, TBI-induced excessive glutamate release and accumulation lead to the sustained activation of NMDA and AMPA receptors. This triggers an excitotoxic cascade involving calcium overload, mitochondrial dysfunction, increased reactive oxygen species (ROS) production, and neuronal apoptosis (Sheldon and Robinson, 2007; Guerriero et al., 2015). Furthermore, dynamic monitoring of cerebrospinal fluid (CSF) cytokines in infants with TBIRD revealed that interleukin-6 (IL-6) levels increased more than 50-fold above baseline on day 3 post-injury, while serum IL-6 remained unchanged (Inoue et al., 2014). This compartmentalized elevation suggests a potential neuroprotective role for endogenous CNS IL-6 in response to excitotoxic injury (Ali et al., 2000). Collectively, current evidence implicates excitotoxicity and associated metabolic disturbances as critical mechanisms in the pathogenesis of TBIRD, providing a theoretical basis for early identification, prognostic assessment, and targeted intervention.

The clinical course of TBIRD exhibits distinct time-dependent characteristics. Patients typically present with acute impaired consciousness or seizures 1–2 days post-injury, followed by delayed neurological deterioration within 3–7 days. MRI demonstrates delayed restricted diffusion in the subcortical white matter, manifesting as the characteristic “bright tree appearance,” which typically emerges 2–10 days following trauma (Takase et al., 2018). In the present case, the patient exhibited immediate consciousness disturbance following the brain injury. A cranial CT scan revealed an acute right fronto-temporo-parietal subdural hematoma with midline shift. On day 3, an MRI revealed the typical “bright tree” signal, after which the patient developed status epilepticus. This clinical evolution and radiological presentation align with previous findings (Takase et al., 2018; Hori et al., 2023). Notably, previous literature reports that TBIRD occurs exclusively in patients under 1.5 years of age (Hori et al., 2023; Inoue et al., 2014; Kaneko et al., 2023; Nishiyama et al., 2014). In contrast, the current patient was 2 years old, making this one of the oldest reported cases of TBIRD to date. Furthermore, the fall occurred from a height of only approximately 50 cm, without significant brain contusion or diffuse hemorrhage. This suggests that the pathogenesis of TBIRD does not depend solely on the severity of mechanical trauma but may be closely linked to metabolic abnormalities and excitotoxicity. A limitation of this study, however, is the lack of magnetic resonance spectroscopy (MRS) data, which prevented the direct assessment of dynamic changes in glutamate and glutamine levels.

Currently, systematic research and evidence-based guidelines for the treatment of TBIRD are lacking. However, as previous studies have suggested, TBIRD appears to share similarities with AESD, and magnetic resonance spectroscopy findings indicate that excitotoxicity may be one of its key pathogenic mechanisms. In the treatment of AESD, previous studies have shown that early administration of vitamin B6 may improve the clinical course of acute encephalopathy. Mechanistically, Vitamin B6 serves as an essential coenzyme for glutamate decarboxylase, facilitating the conversion of glutamate into the inhibitory neurotransmitter *γ*-aminobutyric acid (GABA), thereby mitigating excitotoxicity induced by excessive glutamate accumulation within the central nervous system (Fukui et al., 2019). Therefore, after the patient was diagnosed with TBIRD, vitamin B6 was added to the treatment regimen on the basis of antiepileptic therapy. Vitamin B6 may help maintain the balance between excitatory and inhibitory neurotransmission, potentially preventing the development of TBIRD and improving patient outcomes. However, this hypothesis remains to be validated through further clinical and experimental studies. Furthermore, numerous studies have confirmed a significant elevation in extracellular glutamate concentrations following TBI (Chamoun et al., 2010; Goodrich et al., 2013), indicating that aberrant excitatory amino acid metabolism plays a critical role in secondary brain injury. Consequently, pharmacological agents—such as excitatory amino acid receptor antagonists or glutamate release inhibitors—may improve the prognosis of TBI and TBIRD patients by attenuating glutamate-mediated neurotoxicity. Nevertheless, the efficacy and safety of these interventions require rigorous verification via systematic basic and clinical research. Future investigations should prioritize the early identification of high-risk populations and the development of precision interventions targeting excitotoxicity and energy metabolic dysregulation, thereby offering novel strategies for the prevention and management of TBIRD.

## Data Availability

The original contributions presented in the study are included in the article/supplementary material, further inquiries can be directed to the corresponding authors.

## References

[ref1] Ali C. Nicole O. Docagne F. Lesne S. MacKenzie E. T. Nouvelot A. . (2000). Ischemia-induced interleukin-6 as a potential endogenous neuroprotective cytokine against NMDA receptor-mediated excitotoxicity in the brain. J. Cereb. Blood Flow Metab. 20, 956–966. doi: 10.1097/00004647-200006000-00008, 10894179

[ref2] Bell J. D. Park E. Ai J. Baker A. J. (2009). PICK1-mediated GluR2 endocytosis contributes to cellular injury after neuronal trauma. Cell Death Differ. 16, 1665–1680. doi: 10.1038/cdd.2009.106, 19644508

[ref3] Chamoun R. Suki D. Gopinath S. P. Goodman J. C. Robertson C. (2010). Role of extracellular glutamate measured by cerebral microdialysis in severe traumatic brain injury. J. Neurosurg. 113, 564–570. doi: 10.3171/2009.12.JNS09689, 20113156 PMC3464461

[ref4] Fukui K. O. Kubota M. Terashima H. Ishiguro A. Kashii H. (2019). Early administration of vitamins B1 and B6 and l-carnitine prevents a second attack of acute encephalopathy with biphasic seizures and late reduced diffusion: a case control study. Brain Dev. 41, 618–624. doi: 10.1016/j.braindev.2019.02.015, 30862409

[ref5] Goodrich G. S. Kabakov A. Y. Hameed M. Q. Dhamne S. C. Rosenberg P. A. Rotenberg A. (2013). Ceftriaxone treatment after traumatic brain injury restores expression of the glutamate transporter, GLT-1, reduces regional gliosis, and reduces post-traumatic seizures in the rat. J. Neurotrauma 30, 1434–1441. doi: 10.1089/neu.2012.2712, 23510201 PMC3741415

[ref6] Guerriero R. M. Giza C. C. Rotenberg A. (2015). Glutamate and GABA imbalance following traumatic brain injury. Curr. Neurol. Neurosci. Rep. 15:27. doi: 10.1007/s11910-015-0545-1, 25796572 PMC4640931

[ref7] Hoffe B. Holahan M. R. (2022). Hyperacute Excitotoxic mechanisms and synaptic dysfunction involved in traumatic brain injury. Front. Mol. Neurosci. 15:831825. doi: 10.3389/fnmol.2022.831825, 35283730 PMC8907921

[ref8] Hori E. Akai T. Maruyama K. Saito Y. Taneichi H. Kuroda S. (2023). Acute subdural hematoma in an infant with a biphasic clinical course and late reduced diffusion. Surg. Neurol. Int. 14:442. doi: 10.25259/SNI_703_2023, 38213461 PMC10783695

[ref9] Inoue H. Hasegawa S. Kajimoto M. Matsushige T. Ichiyama T. (2014). Traumatic head injury mimicking acute encephalopathy with biphasic seizures and late reduced diffusion. Pediatr. Int. 56, e58–e61. doi: 10.1111/ped.12411, 25336011

[ref10] Kaneko N. Nishizawa H. Fujimoto J. Nanao T. Kimura Y. Owada G. (2023). An infantile traumatic brain injury with a bright tree appearance detected before the late seizure. Childs Nerv. Syst. 39, 285–288. doi: 10.1007/s00381-022-05589-2, 35739288

[ref11] Kawano A. Sakurai Y. Ishii K. Yokohari H. Nagura M. Uchida Y. . (2025). Clinical characteristics of infantile traumatic brain injury with a biphasic clinical course and late reduced diffusion among pediatric patients with traumatic brain injury. Surg. Neurol. Int. 16:287. doi: 10.25259/SNI_194_2025, 40837317 PMC12361708

[ref12] Nagai K. Kawano G. Sakaguchi H. Yokochi T. Eshima N. Matsuishi T. (2025). Post-encephalitic epilepsy in patients with acute encephalopathy with biphasic seizures and late reduced diffusion. Front. Neurol. 16:1568566. doi: 10.3389/fneur.2025.1568566, 40765618 PMC12321538

[ref13] Nishiyama M. Fujita K. Maruyama A. Nagase H. (2014). Two cases of traumatic head injury mimicking acute encephalopathy with biphasic seizures and late reduced diffusion. Brain Dev. 36, 928–931. doi: 10.1016/j.braindev.2013.12.006, 24411946

[ref14] Paz J. T. Huguenard J. R. (2015). Microcircuits and their interactions in epilepsy: is the focus out of focus? Nat. Neurosci. 18, 351–359. doi: 10.1038/nn.3950, 25710837 PMC4561622

[ref15] Popescu C. Anghelescu A. Daia C. Onose G. (2015). Actual data on epidemiological evolution and prevention endeavours regarding traumatic brain injury. J. Med. Life 8, 272–277, 26351526 PMC4556905

[ref16] Sheldon A. L. Robinson M. B. (2007). The role of glutamate transporters in neurodegenerative diseases and potential opportunities for intervention. Neurochem. Int. 51, 333–355. doi: 10.1016/j.neuint.2007.03.012, 17517448 PMC2075474

[ref17] Takanashi J. Mizuguchi M. Terai M. Barkovich A. J. (2015). Disrupted glutamate-glutamine cycle in acute encephalopathy with biphasic seizures and late reduced diffusion. Neuroradiology 57, 1163–1168. doi: 10.1007/s00234-015-1573-x, 26298309

[ref18] Takase N. Igarashi N. Taneichi H. Yasukawa K. Honda T. Hamada H. . (2018). Infantile traumatic brain injury with a biphasic clinical course and late reduced diffusion. J. Neurol. Sci. 390, 63–66. doi: 10.1016/j.jns.2018.04.010, 29801909

[ref19] Yasukohchi M. Omata T. Ochiai K. Sano K. Murofushi Y. Kimura S. . (2024a). Factors influencing the development of infantile traumatic brain injury with a biphasic clinical course and late reduced diffusion. J. Neurol. Sci. 457:122904. doi: 10.1016/j.jns.2024.122904, 38290378

[ref20] Yasukohchi M. Omata T. Ochiai K. Sano K. Murofushi Y. Kimura S. . (2024b). Pathomechanism of infantile traumatic brain injury with a biphasic clinical course and late reduced diffusion evaluated by MR spectroscopy. J. Neurol. Sci. 466:123228. doi: 10.1016/j.jns.2024.123228, 39278172

